# Development and validation of a higher-throughput, automated FAMA assay for varicella-zoster virus antibody detection, offering a scalable alternative to the classic 12-well slide method

**DOI:** 10.1128/spectrum.01962-26

**Published:** 2026-09-23

**Authors:** Yaru Quan, Xian Yang, Ping Qiu, Zhen Chen, Jinle Han, Jiaojiao Nie, Xingchao Geng

**Affiliations:** 1 Division of Respiratory Virus Vaccines, National Institutes for Food and Drug Control 12540, Beijing, China; 2 Beijing Wantai Biological Pharmacy Enterprise Co., Ltd., Beijing, China.; National Microbiology Laboratory, Winnipeg, Manitoba, Canada

**Keywords:** varicella-zoster virus, fluorescent antibody to membrane antigen assay, higher-throughput serological assay, vaccine immunogenicity, permanent traceability, method validation

## Abstract

The classical fluorescent antibody to membrane antigen (FAMA) slide assay is the gold standard for varicella-zoster virus (VZV) antibody detection, yet it suffers from low throughput, subjective interpretation, labor-intensive operation, and poor data traceability. To address these limitations, a 96-well plate FAMA assay was developed and preliminarily validated based on the classical FAMA principle for quantitative VZV antibody detection. VZV-infected MRC-5 cells were used as substrate to prepare higher-throughput FAMA plates, and clinical samples were tested using an automated system. Cut-off determination, comprehensive method validation, and consistency comparison with the classical FAMA slide assay were performed. The established higher-throughput FAMA assay demonstrated comparable performance to the classical method in terms of signal specificity and sensitivity under the tested conditions while improving throughput and reducing turnaround time. Automated interpretation and storage ensured objective and traceable results, with an overall concordance of 88% (*r* = 0.886) with the classical FAMA slide assay. This pilot study suggests that the higher-throughput FAMA assay has the potential to serve as an alternative to the classical FAMA slide assay for large-scale VZV serological surveys and varicella/zoster vaccine immunogenicity evaluation, providing a more efficient and objective tool for vaccine and drug development.

**IMPORTANCE:**

The gold-standard antibody test for varicella-zoster virus (VZV), the membrane antigen fluorescent antibody (FAMA) method, is labor-intensive, subjective, and poorly suited for large-scale use. To overcome these limitations, we developed and preliminarily validated a higher-throughput, automated 96-well FAMA method. This novel approach retains good sensitivity and specificity while enabling rapid, objective, and traceable results. Pilot validation demonstrated 88% agreement (*r* = 0.886) with the classic method, with acceptable performance across specificity, selectivity, precision, linearity, stability, and durability. This scalable method shows potential to meet clinical requirements and could serve as an alternative to manual procedures for large-scale antibody detection, supporting more efficient vaccine development and immune monitoring against VZV.

## INTRODUCTION

Varicella represents an acute respiratory infectious disease triggered by varicella-zoster virus (VZV), which predominantly circulates among children, with over 90% of individuals acquiring natural infection prior to puberty (1, 2). The 2019 Global Burden of Disease Study estimated around 84 million global varicella cases and 15,000 attributable deaths each year (3). Within China’s national notifiable infectious disease surveillance system, varicella stands as the third most common vaccine-preventable disease after tuberculosis and influenza (4). Upon primary infection, VZV establishes lifelong latency in neuronal ganglia. Decades later, viral reactivation can induce herpes zoster, severely deteriorating the quality of life in elderly individuals (5–8).

VZV is an enveloped spherical DNA virus. Its envelope glycoproteins, particularly gE and gB, are essential for eliciting protective humoral immunity. Notably, recombinant gE has been utilized as the pivotal antigen for the licensed herpes zoster vaccine Shingrix (6, 9–12). Vaccination constitutes the most effective intervention against VZV infection (13). As of 2024, 56 countries worldwide have incorporated varicella vaccines into routine childhood immunization schedules (14), covering multiple monovalent varicella vaccines and two types of measles-mumps-rubella-varicella combination vaccines, among which four monovalent varicella vaccines have achieved World Health Organization (WHO) prequalification (15). Immunogenicity assessment is indispensable throughout vaccine development and head-to-head comparative studies; nevertheless, methodological heterogeneity and inherent technical bottlenecks substantially restrict the advancement of relevant investigations.

Currently available laboratory approaches for VZV antibody detection include the fluorescent antibody to membrane antigen (FAMA) assay, immune adherence hemagglutination assay, neutralization test, and enzyme-linked immunosorbent assay (ELISA) (9). Of these methodologies, only FAMA and ELISA have been widely implemented in clinical settings. Regarded as the gold standard, the conventional FAMA slide assay exhibits excellent correlation with clinical protection and has been broadly employed for immunogenicity evaluation of varicella and herpes zoster vaccines (16–19). The US Centers for Disease Control and Prevention (CDC)’s Advisory Committee on Immunization Practices (ACIP) has recognized a FAMA titer ≥1:4 as a serological correlate of protection at 16 weeks post-vaccination (17). In China, FAMA remains a benchmark assay for clinical evaluation of VZV vaccines, supporting batch-to-batch consistency verification, long-term immunogenicity persistence monitoring, and cross-manufacturer comparative analysis (20–23). Nevertheless, the classical FAMA slide assay is labor-intensive and entirely manual, accompanied by low testing throughput, subjective endpoint interpretation, cumbersome operational workflows, unsatisfactory inter-laboratory reproducibility, and inadequate data traceability (9). Such shortcomings severely limit its applicability to large-scale clinical specimen screening and multi-vaccine comparative assessment.

To address these unmet needs and provide a universal immunogenicity evaluation tool for clinical trials of varicella vaccines (primary prevention) and zoster vaccines (including live attenuated and recombinant subunit vaccines), we established a higher-throughput, automated, standardized, and data-integrated VZV antibody detection platform based on the classic FAMA principle. Preliminary validation results demonstrated that this newly developed assay showed comparable sensitivity and specificity of conventional FAMA while markedly increasing throughput, shortening assay turnaround time, and enabling objective, traceable data archiving, showing potential to satisfy clinical diagnostic and regulatory requirements. The higher-throughput FAMA achieved an overall concordance of 88% and a correlation coefficient of 0.886 relative to the traditional slide-based method. This optimized approach has the potential to serve as an alternative for large-scale VZV serosurveillance and immunogenicity evaluation of varicella-zoster vaccines and may accelerate the research and translational development of VZV vaccines and related antiviral therapeutics.

## MATERIALS AND METHODS

### Preparation of higher-throughput FAMA plates

The VZV strain was preserved by Beijing Wantai Biological Pharmacy Enterprise Co., Ltd., and MRC-5 cells were purchased from the American Type Culture Collection (ATCC). The V-Oka strain of VZV was inoculated into well-maintained MRC-5 cells at a multiplicity of infection (MOI) of 0.01. Following incubation until 50–100% cytopathic effect (CPE) was observed, the culture supernatant was aspirated and discarded. The cells were digested with trypsin and centrifuged at 300 × *g* for 5 min, and the supernatant was discarded. The cell density was adjusted to 10,000–30,000 cells per well using complete culture medium, and 100 μL of the cell suspension was dispensed into each well of a 96-well cell culture plate. Fixatives, including acetone, glutaraldehyde, or formaldehyde, were added individually, and the plates were fixed at room temperature for 30 min. After fixation, the plates were washed five times with phosphate-buffered saline (PBS) supplemented with 0.5% Tween 80, vacuum-sealed, and stored at ≤ −60℃. Random sampling was performed in triplicate, and Hoechst nuclear staining reagent (Beyotime Biotechnology, Shanghai, China) was used to stain cells for the evaluation of inter-plate homogeneity.

### Optimization of clinical sample detection method

Clinical sera were inactivated at 56℃ for 30 min, then serially diluted twofold with PBS supplemented with different additives (e.g., sodium chloride, sucrose, mannitol, bovine serum albumin, human serum albumin, urea, heparin, and ammonium sulfate). The diluted sera were dispensed into the higher-throughput FAMA plates at 100 μL per well and incubated at 37℃ for 30, 60, or 70 min. After incubation, the plates were washed five times with an automated plate washer, followed by the addition of a goat anti-human IgG-fluorescein isothiocyanate (FITC) antibody (Sigma-Aldrich, St. Louis, MO, USA) and re-incubation at 37℃ for 30 or 60 min. The liquid was aspirated and discarded, and the plates were patted dry. Hoechst nuclear staining reagent was added and incubated at room temperature for 5 min, after which the plates were washed again before glycerol was added. Image acquisition and automated analysis were performed using several instruments: a conventional fluorescence microscope (Ti2-D-PD, Nikon, Japan) for small-scale morphological observation, a high-content microscope (Molecular Devices, San Jose, CA, USA) for automated higher-throughput single-cell analysis, an enzyme-linked immunospot analyzer (sPot, AID, Germany), and a Celigo microscope (200-BFFL-5C, Revvity, MA, USA) for rapid cell counting. Unless otherwise specified, all results shown were obtained using the high-content microscope. The antibody titer was defined as the highest dilution showing a complete fluorescent ring on the cell membrane according to international FAMA guidelines. The international reference standard NIBSC W1044 (VZV FAMA standard) was included in each experimental run. The run was considered valid only if the standard’s measured titer fell within the supplier-defined acceptable range; this allowed batch-to-batch calibration and assessment of inter-assay reproducibility.

### FAMA slide assay

Clinical sera were inactivated at 56℃ for 30 min and serially diluted twofold with PBS. The diluted sera were dispensed onto FAMA slides at 25 μL per well and incubated at 37℃ for 30 min. After incubation, the slides were washed on a shaker at 140 rpm for 6 min, then allowed to stand for 4 min, and this process was repeated three times. Finally, the slides were repeatedly immersed in purified water three times and air-dried at room temperature. Goat anti-human IgG-FITC antibody was added, and after re-incubation at 37℃ for 30 min, the slides were washed using the same method. After air-drying at room temperature, glycerol was added, and the slides were covered with coverslips for mounting. Serum antibody titers were observed and recorded using a fluorescence microscope.

### Cut-off value determination

A total of 106 serum samples negative for VZV antibodies by FAMA slide assay were identified during baseline screening of participants enrolled in a phase III clinical trial (approval no. 2023-002-02) at Hubei Provincial Center for Disease Control and Prevention and used in this study under the same ethical approval. Three independent experimenters performed detections using three different batches of reagents according to the method established in the Optimization of clinical sample detection method section. The detection data were analyzed using Minitab Statistical Software 21 (Minitab, LLC, PA, USA). First, the Anderson-Darling test was used to assess the normality of the detection data. If the data were normally distributed, the upper limit of the 95% confidence interval (CI) was calculated as the cut-off value; if not normally distributed, the detection data were sorted in ascending order, and the one-sided 95th percentile was used as the cut-off value.

### Method validation

To fully verify the performance of the method and meet clinical trial requirements, with reference to ICH M10: Bioanalytical Method Validation and Study Sample Analysis (24) and Technical Guidelines for the Research of Antibody Analysis Methods in Vaccine Clinical Trials (25), comprehensive validation of specificity, selectivity, relative accuracy and precision, linearity, stability, and robustness was performed on the method established in the Optimization of clinical sample detection method section based on the concepts of quality by design (QbD) and analytical method lifecycle management.

Specificity evaluation was conducted using four distinct sample combinations consisting of negative reference samples, 1:1 mixtures of negative and positive references, 1:1 mixtures of VZV virus solution and negative references, and 1:1 mixtures of VZV virus solution and positive references to systematically assess matrix effects and pathogen-specific detection performance. Negative reference serum samples were obtained from baseline screening of a phase III varicella vaccine trial (approval no. 2023-002-02) at Hubei Provincial Center for Disease Control and Prevention. Positive reference serum samples were sourced from a separate varicella and herpes zoster vaccine trial (ChiCTR20252239), in which post-vaccination serum samples tested positive by the FAMA slide assay. The validation criteria required consistent negative results for negative reference samples and positive results for positive reference samples, with no detectable interference of exogenous VZV virus on the accuracy of detection outcomes.

Method selectivity was assessed using one clinically derived hemolyzed serum sample and one hyperlipidemic serum sample. Experimental positive samples containing 5% hemolysis and 2 mM triglycerides were prepared by blending qualified positive references with the hemolytic and hyperlipidemic sera, respectively. Independent detection of normal, hemolyzed, and hyperlipidemic sera was also performed to verify the anti-interference capability of the assay. The acceptance criterion stipulated that hemolysis and hyperlipidemia at the tested concentrations should not induce observable deviations in detection results.

A nested experimental design was adopted for the validation of relative accuracy and precision. Serum samples with high (titers 8,192 and 2,048), medium (512 and 256), and low (32 and 16) VZV antibody titers were selected as test objects. Two independent operators conducted duplicate detections at four different time points using three batches of reagents, generating 48 replicate data points for each titer level. The predefined acceptance thresholds were set as follows: the relative bias and geometric coefficient of variation (GCV) for samples near the limit of detection should not exceed 25 and 20%, respectively; samples at other titer levels should have a relative bias below 20% and a GCV below 15%.

Linearity was evaluated via serial dilution of high-titer positive serum samples. The actual antibody titer of each diluted sample was measured and linearly regressed against the theoretical titer values. A coefficient of determination (*R*²) of no less than 0.980 was defined as the qualified standard for favorable linear correlation.

Stability assessment included thermal accelerated stability and freeze-thaw stability verification using the working reference standard (calibrated with the international reference standard NIBSC W1044). Nine aliquots of this standard were distributed, three per condition (2–8℃ storage, room temperature incubation, and freeze-thaw cycles). For thermal stability testing, detection reagents and reference samples were stored at 2–8℃ for 0 to 5 days or placed at room temperature (21–25℃) for 0 to 8 h prior to detection. For freeze-thaw stability evaluation, reference samples were subjected to zero to five repeated freeze-thaw cycles. The stability acceptance criteria required that all measured results deviated from the theoretical values by no more than one dilution fold, with no obvious declining trend in detection performance.

Robustness was verified by deliberately introducing minor variations to routine experimental parameters. Reference samples at two titer levels—a high titer of 256 and a low titer of 32—were used. For each titer level, aliquots were assigned to four altered conditions: 2 aliquots for 6-h room-temperature placement of detection reagents, 3 aliquots for ±10 min deviation in sample incubation duration, 5 aliquots for ±10 min deviation in antibody incubation duration, and 4 aliquots for a 7-day delay in result collection. Thus, a total of 14 aliquots were tested per titer level under altered conditions (28 aliquots in total across both levels). The assay was considered robust if all test results differed from the theoretical values by no more than one dilution fold.

### Method consistency study

Pre-vaccination and post-vaccination sera were collected from a phase III clinical trial (ChiCTR20252239) of varicella/herpes zoster vaccines. A total of 380 pre-vaccination serum samples were collected from 380 individual participants prior to vaccination, and 610 post-vaccination serum samples were collected from 610 individual participants at 42 days post-vaccination. It should be noted that the pre- and post-vaccination samples were not from fully matched donors, and the sample numbers are reported as total independent samples. Parallel detections were performed using the higher-throughput FAMA method (Optimization of clinical sample detection method) and the FAMA slide assay (FAMA slide assay). Spearman correlation analysis was performed on the detection data using Minitab Statistical Software 21 (Minitab, LLC, PA, USA), and the negative, positive, and overall concordance rates were calculated to evaluate the consistency between the two methods.

## RESULTS

### Establishment and optimization of a higher-throughput FAMA assay

#### Workflow overview

**Fig 1:**
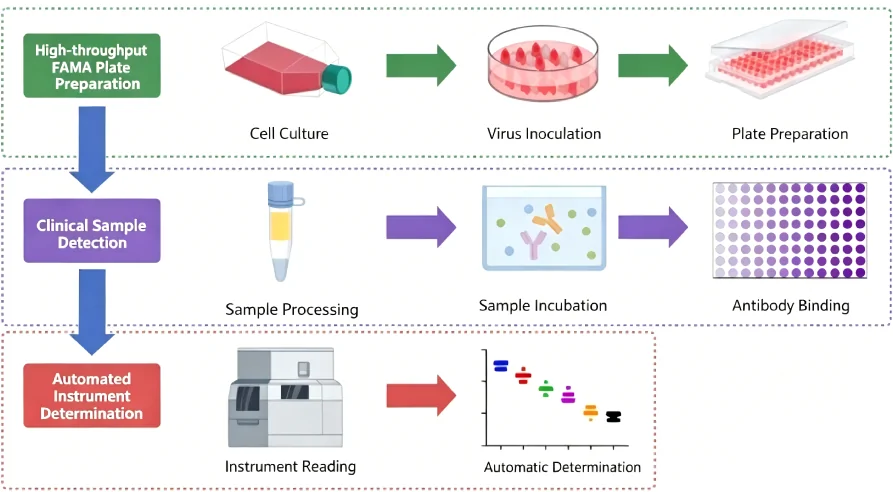
Workflow of the higher-throughput FAMA assay. The assay consists of three major procedures: higher-throughput FAMA plate preparation, clinical sample testing, and automated instrumental result interpretation.

The workflow consisted of three key procedures: higher-throughput FAMA plate preparation, clinical sample testing, and automated instrumental interpretation (Fig. 1). Compared with the traditional glass-slide FAMA method, this assay is compatible with multichannel pipettes, automated liquid handling stations, and microplate washers, which increased detection throughput and efficiency by over 70%. Specifically, the hands-on time for sample loading, washing, and image acquisition/analysis was reduced from approximately 170 to 50 min for 100 samples, with the imaging/analysis step requiring zero hands-on time due to full automation using a robotic arm and a high-content microscope. Integrated with image acquisition and automated counting, the conventional semi-quantitative FAMA assay was converted into an automated platform that outputs continuous and precise spot counts, supporting statistical analysis among different groups and batches.

#### Optimization of higher-throughput FAMA plate preparation

Key parameters, including VZV cytopathic severity, cell seeding density, and fixation conditions, were systematically optimized.

**Fig 2:**
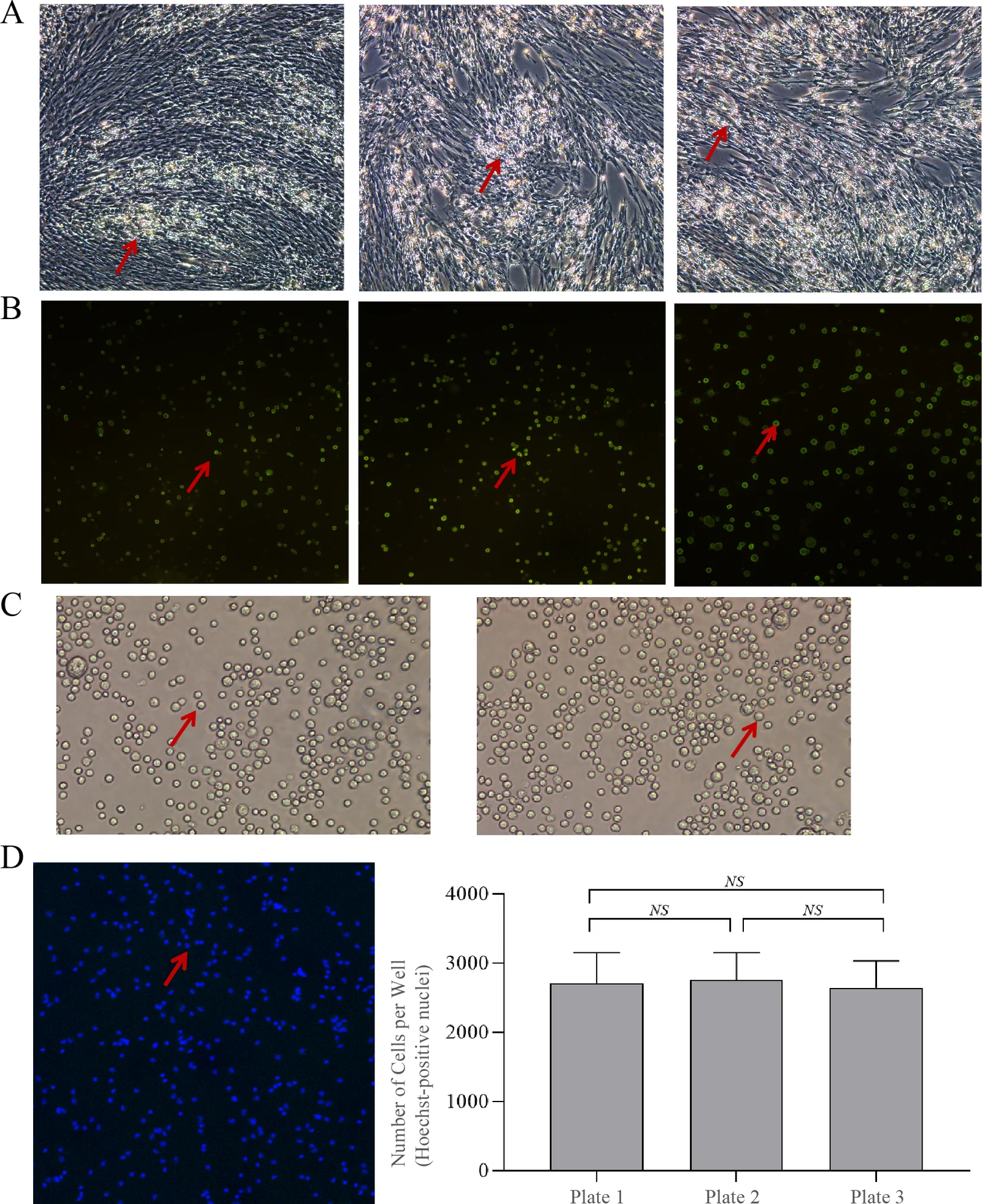
Optimization of preparation conditions for higher-throughput FAMA microplates. (**A**) Dynamic progression of VZV-induced CPE. Red arrows indicate typical cytopathic cells. Representative images showing CPE rates of approximately 40% (left), 60% (middle), and 80% (right), acquired at sequential time points during incubation of VZV-infected MRC-5 monolayers. CPE severity increased progressively over time. (**B**) Effect of CPE severity on fluorescent signal performance. Red arrows mark positive immunofluorescent signals. FAMA microplates prepared at a CPE rate below 60% (left), above 60% (middle), and conventional glass-slide FAMA results (right) are presented. A CPE rate over 60% yielded brighter positive signals and markedly improved detection sensitivity. Notably, the signal morphology of microplate-based FAMA was highly consistent with that of traditional glass-slide FAMA, retaining the intrinsic characteristics of *in situ* immunofluorescence. (**C**) Optimization of cell seeding density and fixative selection. Red arrows represent fixed intact cells. At a seeding density of 10,000–20,000 cells per well, MRC-5 cells formed uniform monolayers without overlapping or aggregation. Parallel fixation with glutaraldehyde (left) and formaldehyde (right) both preserved complete cell morphology and satisfactory antigen localization. (**D**) Homogeneity assessment of optimized FAMA microplates. Left: Hoechst staining (red arrows) showing even cell distribution without stacking, consistent with post-fixation morphology. Right: quantitative counting of Hoechst-positive nuclei from three independent plates (*n* = 10 wells per plate). Data are presented as mean ± SD, and excellent intra-plate and inter-plate homogeneity is verified with no significant difference among groups (*P* > 0.05). NS indicates no statistically significant difference.

First, the degree of the CPE was examined. The CPE of VZV-infected monolayer MRC-5 cells increased with the incubation time (Fig. 2A). The CPE below 60% resulted in insufficient expression of viral membrane glycoproteins and weak fluorescent signals, while the that above 60% significantly enhanced signal intensity and detection sensitivity (Fig. 2B). Therefore, cells with 60–100% CPE were used for plate preparation to guarantee adequate antigen density for capturing serum-specific antibodies.

Having established the optimal CPE range, we next investigated cell seeding density. Cell seeding density directly affected cell distribution and signal interpretation. Seeding at 30,000 cells per well caused obvious cell stacking and impaired signal localization, whereas a density of 10,000–20,000 cells per well yielded uniform monolayer distribution without overlapping, facilitating instrumental auto-focusing and image analysis. The optimal cell density was determined as 10,000–20,000 cells per well.

Another critical factor was the choice of fixative, as the FAMA assay relies on *in situ* immunofluorescence. The FAMA assay relies on *in situ* immunofluorescence. This requires intact cell morphology and stable retention of viral antigens on cell membranes. Cross-linking fixatives (glutaraldehyde and formaldehyde) formed intermolecular methylene bridges via aldehyde-amino crosslinking, maintaining cell integrity and natural distribution of membrane antigens (Fig. 2C). In contrast, acetone disrupted lipid membranes and cellular structure by protein dehydration and denaturation, making it unsuitable for intact cell fixation.

To rigorously assess the plate-to-plate and well-to-well uniformity under the optimized conditions, we quantified the cell distribution by Hoechst 33342 nuclear staining (Fig. 2D). For each of the three independently fabricated plates, 10 random wells were selected for cell counting. The mean cell numbers per well (±SD) were 2,704 ± 443, 2,757 ± 392, and 2,642 ± 91 for plates 1, 2, and 3, respectively, with corresponding coefficients of variation (CV) of 16.4, 14.2, and 3.4%—all well below the 20% acceptance criterion. A one-way analysis of variance (ANOVA) revealed no statistically significant difference among the three plates (*P* > 0.05), confirming the high reproducibility and uniform cell seeding of the optimized plates.

#### Optimization of clinical sample detection

Nonspecific fluorescence in clinical serum samples interfered with automated spot counting. To identify optimal assay conditions, we first performed semi-quantitative visual screening of sample diluent formulation, incubation time, and detection instruments to reduce background interference.

**Fig 3:**
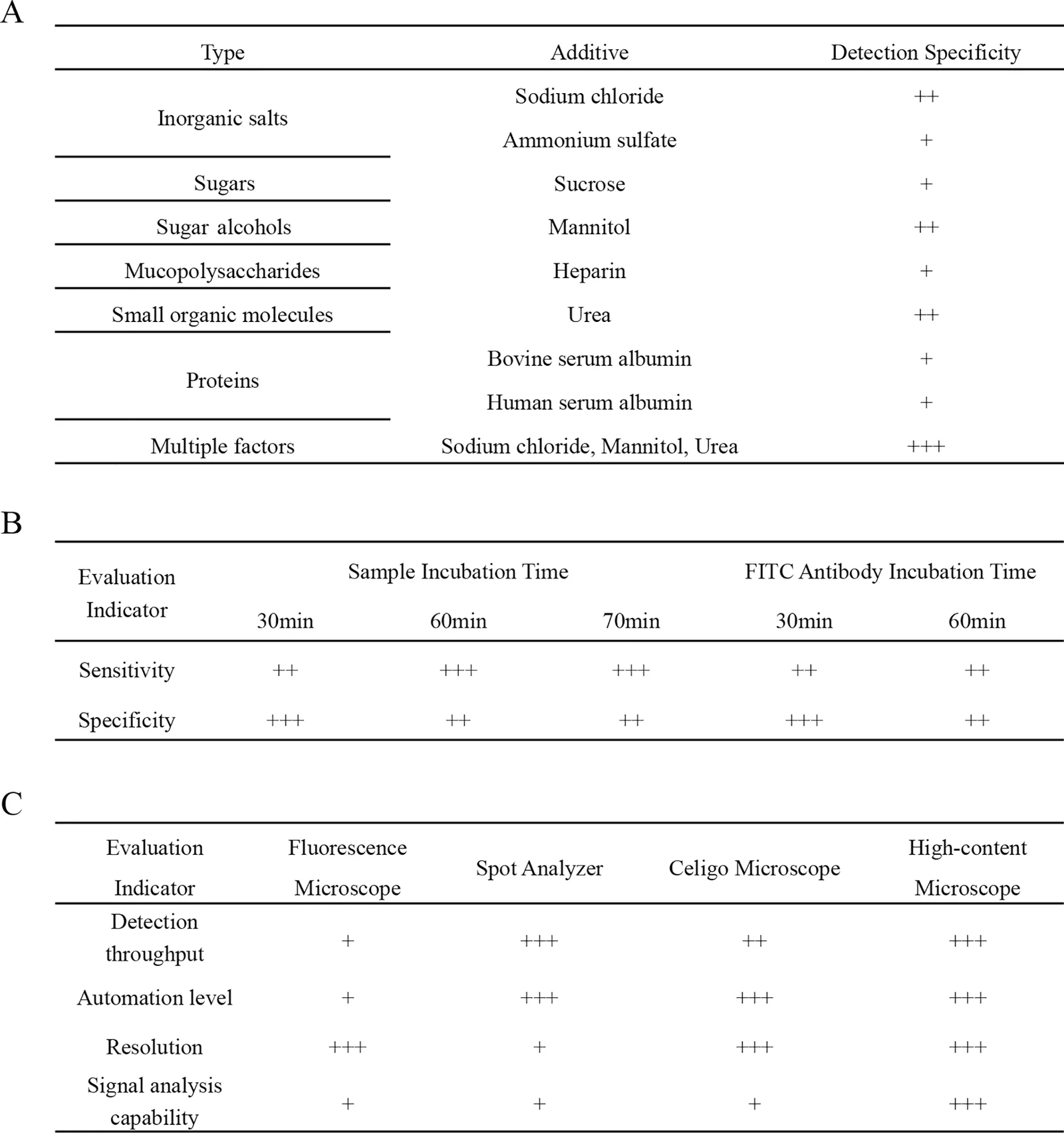
Optimization of experimental conditions for clinical sample detection. Rating scale: +++, excellent; ++, good; +, poor. (**A**) Screening of additive components for sample diluent. Sodium chloride, mannitol, and urea individually improved detection specificity, while combined application exerted a synergistic effect and achieved optimal performance. (**B**) Robustness evaluation of incubation time for samples and FITC-conjugated secondary antibodies. Within 30–70 min for sample incubation and 30–60 min for antibody incubation, the assay maintained stable positive signal intensity and background signal, demonstrating good methodological robustness. (**C**) Comprehensive performance comparison of different detection platforms. Throughput and resolution were primarily determined by hardware configuration; signal analysis capability relied on algorithm software; and automation level was jointly governed by both hardware and software. The high-content imaging system exhibited excellent overall performance. All scoring was performed by two experienced researchers in a double-blind manner.

To evaluate the effect of different additive combinations in sample diluent formulation on detection specificity, we defined relative detection specificity as a semi-quantitative visual score (“+,” “++,” or “+++”) based on the background fluorescence of negative sera, where “+” represents the reference condition (basic diluent), and “++”/ “+++” indicates a progressively cleaner background and a higher specificity. Our results showed that sodium chloride reduced nonspecific binding by shielding electrostatic adsorption; mannitol stabilized protein conformation and inhibited hydrophobic interaction to lower background noise; and urea disrupted weak nonspecific protein interactions by breaking hydrogen bonds. Combined application of the three reagents produced synergistic effects on mitigating serum matrix interference and improving assay specificity, with performance superior to single-component use (Fig. 3A).

To examine the influence of incubation duration on antigen-antibody binding and background accumulation, we visually scored the positive signal intensity and background noise on a semi-quantitative scale (“+,” “++,” and “+++”). Prolonged sample incubation from 30 to 70 min gradually increased the positive signal intensity, indicating enhanced binding of low-affinity antibodies; meanwhile, the background signal showed a slight rise but remained below the predefined positivity threshold within an acceptable range. Incubation with FITC-conjugated secondary antibody for 30 to 60 min resulted in stable positive signal intensity and background signal, confirming favorable assay robustness within the tested time ranges (Fig. 3B).

Four detection platforms were further compared in terms of throughput, automation, resolution, and signal analysis performance, including fluorescence microscope, spot analyzer, Celigo imaging system, and high-content microscope. The high-content microscope exhibited optimal comprehensive performance and was selected as the standard detection platform for subsequent experiments (Fig. 3C).

### Cut-off value establishment

**Fig 4:**
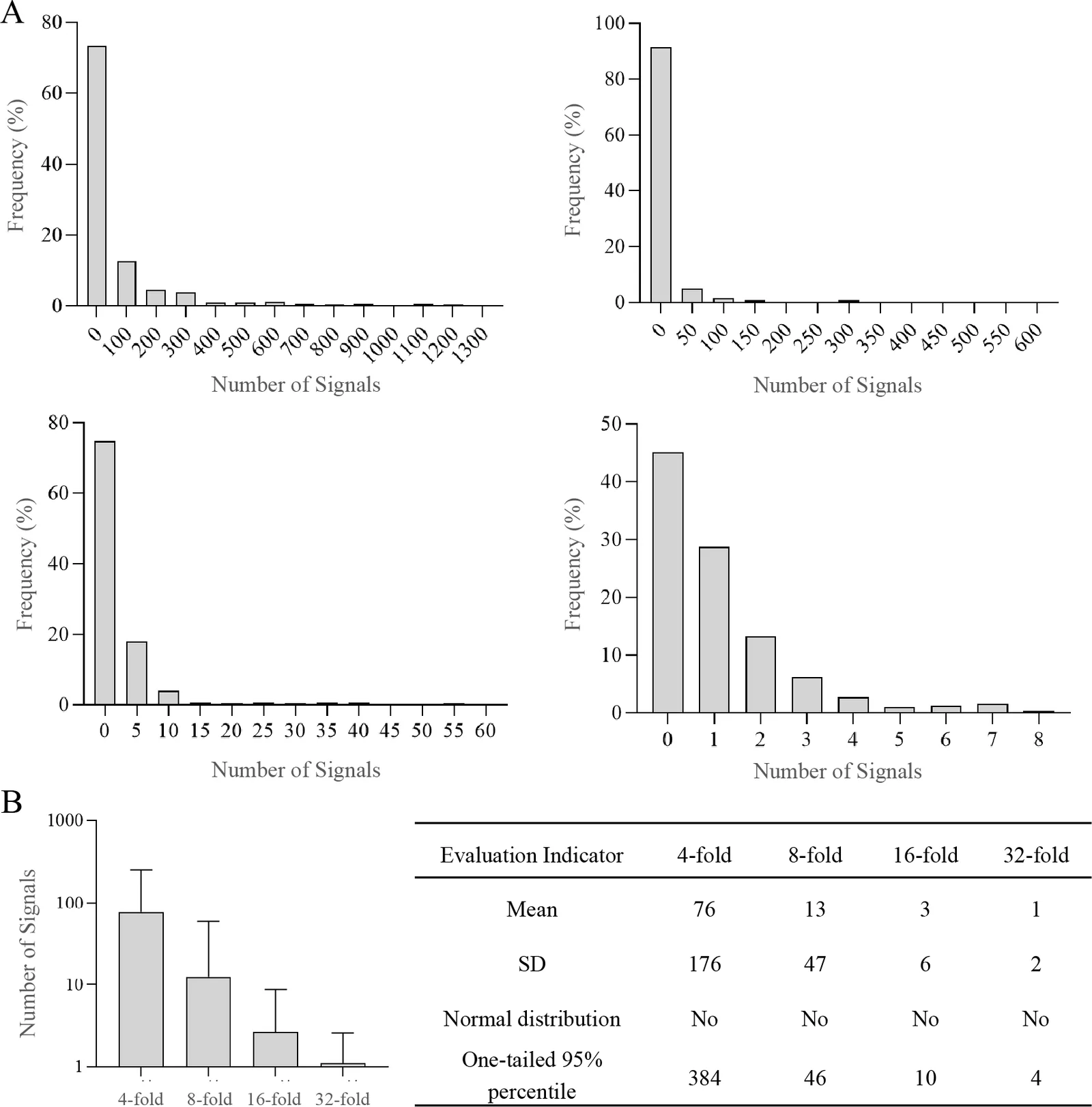
Establishment and optimization of the assay cut-off value. (**A**) Distribution characteristics of background signal counts for negative samples at different dilutions. Four dilution gradients are displayed: 4-fold (upper left), 8-fold (upper right), 16-fold (lower left), and 32-fold (lower right). “Numbers of signals” indicates the number of background fluorescent cells observed in one sample; “Frequency (%)” is the proportion of samples showing a given signal count. All groups exhibited obvious right-skewed distribution accompanied by a small number of high-value outliers. (**B**) Dilution-stratified strategy for cut-off value determination. Background signal levels differed significantly across dilutions and gradually declined to near baseline at 32-fold dilution. Statistical analysis confirmed that negative signal data at all dilutions disobeyed normal distribution. The one-tailed 95% percentile method was, therefore, adopted to define the cut-off value for each dilution gradient independently.

A total of 324 valid data points (from 106 negative serum samples, six of which were tested four times and 100 tested three times) were enrolled for the cut-off value calculation. At serial dilutions ranging from 4- to 32-fold, signal counts presented a typical right-skewed distribution, with most samples concentrated at low values and a small number of high-value outliers (Fig. 4A). The Anderson-Darling normality test further confirmed the non-normal distribution of raw data. A conventional confidence interval method based on normal distribution would overestimate the cut-off value due to outliers, increasing false negatives. Therefore, the distribution-independent one-tailed 95% percentile method was adopted. For each dilution, the cut-off was defined as the 95% percentile of background signal counts from the 324 negative sera; a test sample with a signal count above this cut-off was considered positive at that dilution. Background signal levels differed significantly across dilution folds. Lower dilutions exhibited higher background signals, while the baseline became stable at 32-fold dilution and above. Adoption of a unified cut-off value for all dilutions would increase false positivity at low dilution levels. Therefore, dilution-stratified cut-off values were separately defined for 4-, 8-, 16-, and ≥32-fold dilutions to ensure accurate and reliable result interpretation (Fig. 4B). This dilution-specific cut-off strategy is particularly critical for accurately determining low-titer antibodies, as it avoids both false-positive and false-negative errors near the positivity threshold.

### Method validation

Assay performance was preliminarily validated in terms of specificity, selectivity, accuracy, and precision, linearity, stability, and robustness to assess its potential for clinical sample detection.

**Fig 5:**
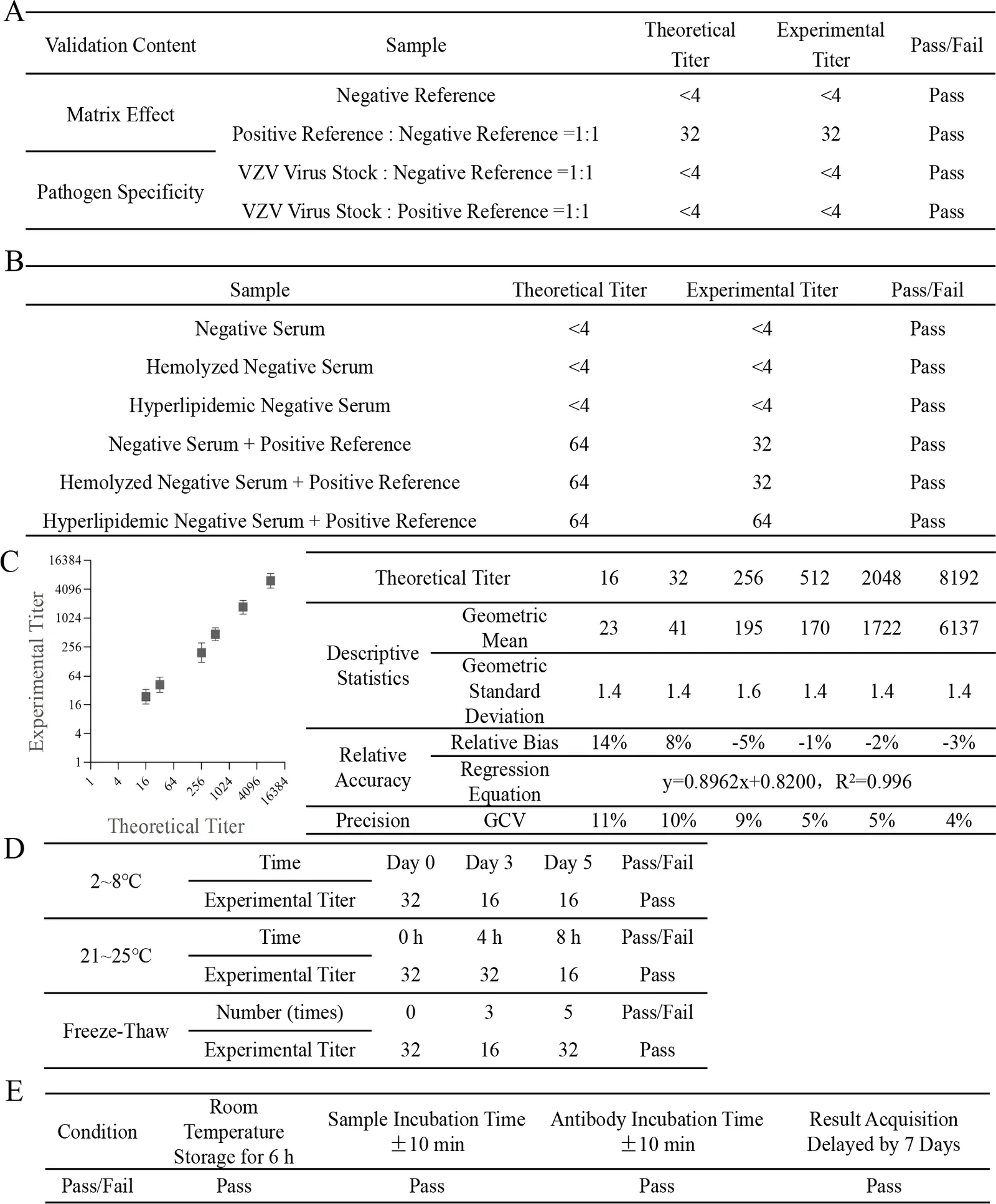
Methodological validation of the higher-throughput FAMA assay. (**A**) Assay specificity. Pre-incubation of the positive reference with VZV virus stock (1:1) resulted in a titer of <4 due to antibody neutralization by viral particles, confirming that the assay specifically detects anti-VZV antibodies without interference from MRC-5 cellular components or other matrix substances. The VZV virus stock used in this experiment had a titer of 1 × 10⁶ TCID₅₀/mL. (**B**) Assay selectivity. Negative and positive serum samples were spiked with 5% hemolyzed blood or 2 mM hyperlipidemic emulsion. All spiked samples yielded titers consistent with theoretical values, indicating minimal interference from common endogenous serum substances. (**C**) Relative accuracy, precision, and linearity. Relative bias and GCV values were both below 15%, meeting the acceptance criteria. Excellent linear correlation was observed across the detection range of 16 to 8,192, with *R*² = 0.996 and no evident hook effect. (**D**) Assay stability. Storage stability at 2–8℃ and thermal acceleration stability at room temperature, as well as freeze–thaw stability, were evaluated for assay reagents and reference samples. No obvious performance attenuation was observed under all tested conditions. (**E**) Assay robustness. Minor variations in ambient placement time, antigen–antibody incubation duration, and image acquisition time did not alter detection performance, indicating favorable operational tolerance for routine experimental deviation.

To evaluate the specificity of the assay, we performed an experiment in which the positive reference serum was mixed with VZV virus stock at a 1:1 ratio prior to testing (Fig. 5A). As expected, the resulting titer dropped from 32 to <4 because the anti-VZV antibodies in the positive reference were pre-neutralized by the viral particles. This neutralization effect confirms that the positive signals detected by the assay are specifically attributable to anti-VZV antibodies, rather than non-specific binding to MRC-5 cellular components or other irrelevant matrix substances. The VZV virus stock was used at a concentration of 1 × 10^6^ TCID_50_/mL in this experiment.

For assay selectivity, no interference was observed using hemolyzed or hyperlipidemic specimens (Fig. 5B). Negative serum samples spiked with 5% hemolyzed blood or 2 mM lipid emulsion, as well as their corresponding positive reference-spiked counterparts, all yielded titers consistent with theoretical values, confirming that common endogenous serum substances do not compromise assay performance.

Relative accuracy and precision were evaluated across different reagent batches, operators, detection time, and replicate tests. Both relative bias and GCV were below 15% (Fig. 5C). High-titer samples showed good accuracy and precision with variations mainly derived from random error. Low-titer samples near the detection limit presented slightly increased bias and variation due to compromised discrimination between weak specific signals and background noise, with variations affected by both random and systematic errors. The established assay exhibited stable accuracy and precision, supporting its potential for reliable laboratory transfer and large-scale sample testing.

Linearity analysis was performed to evaluate the proportional relationship between measured and expected values. A strong linear correlation was observed across the 16- to 8,192-fold dilution range (*R*² = 0.996), and no obvious hook effect was detected within the tested range.

Stability assessment covered reagent storage, sample handling, and repeated freezing and thawing. Assay reagents and clinical samples remained stable for at least 5 days at 2–8℃, tolerated 8 h of room-temperature exposure, and withstood up to five freeze-thaw cycles without performance deterioration (Fig. 5D).

Assay robustness was evaluated by introducing minor fluctuations in experimental parameters. Slight changes in room-temperature placement duration, sample and antibody incubation time, as well as image acquisition time, exerted no significant impact on overall detection performance, allowing acceptable operational tolerance for inter-laboratory method transfer and routine experimental variation (Fig. 5E).

### Method consistency evaluation

**Fig 6:**
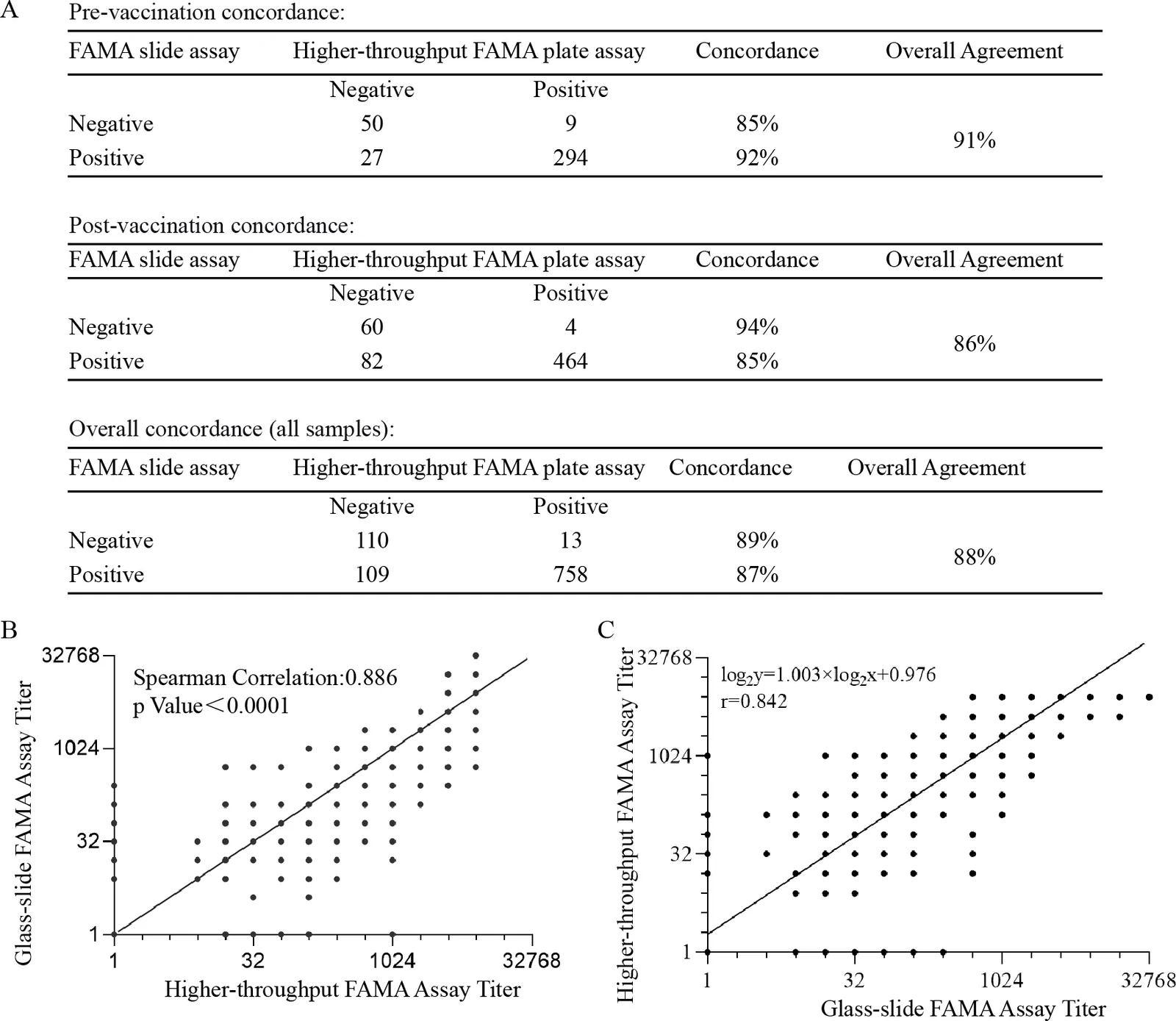
Correlation and consistency between the higher-throughput FAMA plate assay and the conventional glass-slide FAMA assays. (**A**) Contingency tables stratified by vaccination status (pre-, post-, and overall), with negative and positive percent agreements and overall agreement provided in each panel. (**B**) Scatter plot showing titer correlation between the two methods (Spearman’s *r* = 0.886, 95% CI: 0.868–0.902, *n* = 990, *P* < 0.0001). The *P*-value (<0.0001) corresponds to the test of the null hypothesis that the true correlation equals zero, confirming that the observed correlation is statistically significant. (**C**) Passing–Bablok regression of log₂-transformed titers (all titers + 1 prior to transformation). Regression equation: log₂y = 1.003 × log₂x + 0.976; bias = 0.853 (95% CI: 0.720–0.986), relative bias = 13%, indicating constant bias (lower sensitivity of the glass-slide assay) without proportional bias.

Consistency between the established higher-throughput FAMA assay and traditional glass-slide FAMA was evaluated for serological detection applicable to varicella-zoster vaccine clinical samples. As shown in the contingency tables (Fig. 6A), the overall agreement rate between the two methods was 88% (negative: 89%, positive: 87%). Stratified by vaccination status, the pre-vaccination overall agreement was 91% (negative: 85%, positive: 92%), and the post-vaccination overall agreement was 86% (negative: 94%, positive: 85%). The Spearman correlation analysis (Fig. 6B) revealed a strong positive correlation between the two methods (*r* = 0.886, 95% CI: 0.868–0.902, *n* = 990, *P* < 0.0001). To further evaluate the nature of the relationship between the two assays, Passing-Bablok regression was performed on log₂-transformed titers (all titers were added by one prior to transformation to ensure all values > 0). As shown in Fig. 6C, the regression equation was log_₂_y = 1.003 × log_₂_x + 0.976. The slope of 1.003 indicated the absence of proportional bias, whereas the intercept of 0.976 suggested the presence of constant bias. The estimated bias was 0.853 (95% CI: 0.720–0.986), corresponding to a relative bias of 13%. Taken together, these results demonstrate that the two methods exhibit strong correlation and good agreement, with a constant systematic difference that warrants further attention in assay standardization (Fig. 6).

### Optimized protocol for the higher-throughput FAMA assay

Based on the systematic optimization described above, the final locked protocol was established as follows: MRC-5 cells were infected with the V-Oka strain of VZV at an MOI of 0.01 and cultured until the CPE reached >60%. The infected cells were then harvested by trypsinization, centrifuged at 300 × *g* for 5 min, and resuspended in culture medium. The cell density was adjusted to 1 × 10^4^ cells per well, and 100 µL of the cell suspension was seeded into each well of a 96-well plate. After seeding, the cells were fixed with 100 µL/well of formaldehyde at room temperature for 30 min. Following fixation, the plates were washed five times with PBS containing 0.5% Tween 80, then vacuum-sealed and stored at ≤ −60℃ until use.

For the binding assay, test serum samples were serially diluted twofold in serum dilution buffer (PBS containing 0.5% Tween 80 supplemented with 0.2 mM urea and 30 mM mannitol). The diluted sera were added to the fixed cell plates and incubated at 37℃ for 30–70 min. After washing, FITC-conjugated secondary antibody was added and incubated at 37℃ for 30–60 min. The plates were then washed, and Hoechst 33342 nuclear staining reagent was added and incubated at room temperature for 5 minutes. Following a final wash, glycerol was added for plate preservation.

Images were acquired using a Molecular Devices ImageXpress Micro Confocal High-Content System with the following settings: 4× Plan Apo objective, binning 2; Hoechst channel, exposure 10 ms; and FITC channel and exposure 50 ms. Automated analysis was performed using MetaXpress Powercore software (v1.5.0.17) via a three-step sequential pipeline: (i) Hoechst-positive nuclei were identified based on morphometric and intensity criteria to determine total cell count per well; (ii) FITC-positive membrane/cytoplasmic signals were identified using analogous criteria; and (iii) a cell was scored as positive when the membrane-associated FITC intensity exceeded the nuclear FITC intensity by a predefined threshold (≥ 300 relative fluorescence units).

## DISCUSSION

Detection of VZV antibodies is essential for population immunity surveillance and vaccine efficacy evaluation. The conventional glass-slide FAMA assay is regarded as the gold standard for VZV antibody detection (16), with well-documented correlation with clinical protection (17–19). Nevertheless, the conventional FAMA assay relies on manual operation, which inevitably leads to inter-laboratory and inter-batch variability, low detection throughput, subjective result interpretation, and poor traceability of raw data. These limitations hinder its application in large-scale serological testing and comparative evaluation of vaccines from different manufacturers (9). Other alternative methods, such as neutralization assays and ELISA, are constrained by insufficient sensitivity, prolonged experimental cycles, or inadequate methodological validation, thereby restricting their widespread clinical application. Accordingly, optimization of the FAMA assay based on the principle of *in situ* immunofluorescence remains an important research priority, although technical challenges have slowed related progress.

In the present study, the traditional 12-well glass-slide format was redesigned into a 96-well microplate platform compatible with automated laboratory instruments, improving detection efficiency by more than 70%. To address two critical technical bottlenecks, namely, the reproducibility of typical positive signals and the interference of non-specific background fluorescence, key experimental conditions were systematically optimized, including cytopathic severity, cell seeding density, fixative formulation, sample diluent composition, and incubation duration. The optimized protocol yielded positive signal characteristics highly consistent with those of the classical glass-slide FAMA assay while maintaining favorable detection specificity.

To eliminate subjective artificial interpretation and enable full data traceability, a high-content imaging system was integrated into the workflow to achieve automated signal recognition, which could completely replace manual reading. The introduction of analytical tools further transformed the conventional semi-quantitative FAMA assay into an automated semi-quantitative detection platform. Output of continuous and precise spot counts enables more sophisticated statistical comparisons across groups and trials.

From the perspective of methodological standardization, the established assay guarantees the comparability and transferability of detection results through quality control of critical reagents and establishment of reference standards. Intra-plate homogeneity and inter-batch consistency of higher-throughput FAMA plates were rigorously evaluated to stabilize the performance of different preparation batches. Meanwhile, a panel of high-, medium-, and low-titer and negative reference samples was constructed, which can be applied for method transfer among laboratories and routine quality control to ensure the reliability of testing outcomes.

Comprehensive methodological validation was performed in this study, covering specificity, selectivity, relative accuracy, precision, linearity, stability, and robustness. All analytical performance indicators showed preliminary compliance with the technical requirements for serological testing in vaccine clinical trials. Consistency analysis demonstrated that the newly developed higher-throughput FAMA assay exhibited a strong correlation and favorable overall agreement with the conventional glass-slide FAMA assay.

Notably, further large-scale and multicenter correlation studies are still warranted. We also acknowledge that this study provides a pilot evaluation, and additional testing against other herpesviruses, rheumatoid factor, autoantibodies, and anti-reagent antibodies is needed before the assay can be adopted for regulatory clinical trials. Enrollment of clinical trial serum samples from broader regions and more vaccine products will help further validate the robustness of this platform.

In conclusion, this study established a standardized higher-throughput FAMA assay for quantitative detection of VZV antibody titers in varicella and herpes zoster vaccine clinical trials. The proposed method achieves high concordance with the gold-standard glass-slide FAMA assay and integrates multiple superior technical characteristics, including preliminary evidence of acceptable performance, higher detection throughput, objective signal interpretation, complete data traceability, and full automation. Supported by systematic standardization optimization and rigorous full-parameter methodological validation, this novel assay may serve as a pilot alternative standardized approach for VZV serological detection. It provides a reliable technical foundation for clinical trial implementation, regulatory evaluation, routine specimen testing, and long-term immunogenicity surveillance.

## Data Availability

The data that support the findings of this study are available from the corresponding author upon reasonable request.
